# Effect of oxidative stress induced by intracranial iron overload on central pain after spinal cord injury

**DOI:** 10.1186/s13018-017-0526-y

**Published:** 2017-02-08

**Authors:** Fan Xing Meng, Jing Ming Hou, Tian Sheng Sun

**Affiliations:** 1 Third Military Medical University, No. 30 Gaotanyan Street, 400038 Chongqing, China; 2 Department of Orthopedics, Chinese PLA Army General Hospital, Dongcheng District, Nanmencang No. 5, 100700 Beijing, China; 3 Southwest Hospital, Third Military Medical University, No. 30 Gaotanyan Street, 400038 Chongqing, China

**Keywords:** Central pain, Spinal cord injury, Iron, Oxidative stress

## Abstract

**Background:**

Central pain (CP) is a common clinical problem in patients with spinal cord injury (SCI). Recent studies found the pathogenesis of CP was related to the remodeling of the brain. We investigate the roles of iron overload and subsequent oxidative stress in the remodeling of the brain after SCI.

**Methods:**

We established a rat model of central pain after SCI. Rats were divided randomly into four groups: SCI, sham operation, SCI plus deferoxamine (DFX) intervention, and SCI plus nitric oxide synthase (NOS) inhibitor treatment. Pain behavior was observed and thermal pain threshold was measured regularly, and brain levels of iron, transferrin receptor 1 (TfR1), ferritin (Fn), and lactoferrin (Lf), were detected in the different groups 12 weeks after establishment of the model.

**Results:**

Rats demonstrated self-biting behavior after SCI. Furthermore, the latent period of thermal pain was reduced and iron levels in the hind limb sensory area, hippocampus, and thalamus increased after SCI. Iron-regulatory protein (IRP) 1 levels increased in the hind limb sensory area, while Fn levels decreased. TfR1 mRNA levels were also increased and oxidative stress was activated. Oxidative stress could be inhibited by ferric iron chelators and NOS inhibitors.

**Conclusions:**

SCI may cause intracranial iron overload through the NOS–iron-responsive element/IRP pathway, resulting in central pain mediated by the oxidative stress response. Iron chelators and oxidative stress inhibitors can effectively relieve SCI-associated central pain.

## Background

Spinal cord injury (SCI) refers to pathological changes including motor, sensory, and sphincter dysfunction and dystonia, as well as pathological reflexes of the corresponding spinal segment following damage by direct or indirect factors. Previous studies have concentrated on the recovery of motor and sensory functions and have tended to ignore SCI-associated complications. However, central pain is a common complication of SCI, with an incidence as high as 77–86% [1, 2]. Central pain (CP) is a neuropathic syndrome associated with hypersensitivity to pain caused by spinal cord or brain injury. It is often associated with persistent and intolerable lower limb pain [3, 4], which can seriously affect sleep, self-care, rehabilitation, and quality of life. However, the pathogenesis of CP is unclear and only empirical therapy can be applied, leading to pain relief in only 20–30% of patients [5]. CP after SCI has thus become a major problem in the field of spinal injury rehabilitation.

The mechanisms of SCI-associated central pain are currently unclear, and the numerous hypotheses proposed to date have been unable to explain fully the mechanisms responsible for structural and functional remodeling in the brains of patients with CP [6]. However, increasing evidence suggests that structural and functional remodeling of the brain is a key causative factor in SCI-associated CP [7, 8].

Previous studies have shown that intracranial iron overload plays an important role in the development and progression of some central nervous system diseases, such as Alzheimer’s disease, Parkinson’s disease, and cerebral hemorrhage [9–11]. It has also been suggested that oxidative stress injury of neurons is related to iron toxicity via the Fenton reaction; peroxidation may affect neuronal ATPase activity, inhibit calcium influx, mediate inflammation [12, 13], and ultimately lead to neuronal injury or loss [14, 15].

In this study, we investigated the occurrence and mechanisms of intracranial iron overload after SCI and determined if iron overload could further induce oxidative stress injury in a rat model of CP. We also examined the effects of iron chelators and oxidative stress inhibitors, to clarify the effects of iron-induced oxidative stress on neurons and the role of iron overload in oxidative stress injury and central pain after SCI.

## Methods

### Experimental design

Sixty female Sprague–Dawley rats (228.0 ± 16.0 g) were divided randomly into four groups: sham operation group (laminectomy without SCI or other interventions), control group (spinal injury without other interventions), l-arginine group (spinal injury plus intraperitoneal injection of 1.5 mg/kg l-arginine on the first day after the operation, followed by once a week to the end of the experiment), and deferoxamine (DFO) group (spinal injury plus intraperitoneal injection of 100 mg/kg DFO on the first day after the operation, followed by once a week to the end of the experiment).

### Establishment of rat model of SCI

Rats were anesthetized with 10% chloral hydrate (3 ml/kg), and the T9–T11 spinous processes and lamina were exposed under aseptic conditions. T10 laminectomy was performed and the impact area (2–3 mm long) was exposed. The Allen impact method was used to establish the SCI model. Rats in the sham operation group underwent the same laminectomy procedure, but without impact. Rats were fed individually after the operation, and 0.6 g lincomycin was injected intramuscularly within 3 days. Bladder squeezing was conducted to help urination until the rats could urinate independently. In the case of death during feeding, new animals were supplemented.

### Assessment of pain behavior

Spontaneous pain reactions were observed after the operation, including grooming the hind limbs and tail, scratching, licking, and spontaneous screaming.

### Measurement of paw-withdrawal latency (PWL)

PWL was measured at 2, 4, 8, 12, 16, and 24 h after the operation, and an intelligent hot plate was used to measure PWL every day thereafter. The rats’ paws were pressed onto a hot plate at 55 °C, and the withdrawal reaction time was recorded. The procedure was performed three times within a 3-min interval and the final result was the average value.

### Determination of iron levels in the brain cortex

Iron levels in the whole brain and in various brain areas were measured by atomic absorption spectrophotometry. Whole brains or the hind limb sensory areas, thalamus, and hippocampus were collected and weighed, immersed in 20 mmol/1 HEPES buffer (1:20, *w*/*v*), and homogenized. A 30-μl volume of homogenate was mixed with an equal volume of ultrapure nitric acid and digested in a 50 °C water bath for 48 h, then diluted with 3.12 mmol/l nitrate at a 1:10 ratio. A standard curve was prepared using iron solution (50 mg/l) diluted with 5% nitric acid. The blank and sample tubes were measured three times and the absorbance values at 248.3 nm were recorded for analysis.

### Detection of transferrin receptor 1 (TfR1) messenger RNA (mRNA) levels by reverse transcription-polymerase chain reaction (RT-PCR)

Total RNA was extracted from the hind limb sensory cortex, hippocampal, and thalamus tissues of three rats in each group using a TRIzol reagent (Invitrogen, Carlsbad, CA, USA), and 20 μg of RNA was treated with 10 U DNaseI (Takara Bio, Otsu, Japan) for 30 min at 37 °C. cDNA was synthesized using oligo dT primer, and 1 μl was added to the reaction containing 27.5 μl Real-Time PCR Master Mix(TOYOBO), 15 pmol of primers, and 7.5 pmol TaqMan probe, for a total volume of 30 μl. Primers and probes for TfR1 and ferritin (Fn) were designed with Primer Premier 5.0 software and synthesized by Shenggong Biotechnology (Shanghai, China). The sequences were as follows: Tfrc-F, CGT GGA GAC TAC TTC CGT GC and Tfrc-R, GCC AGA GCC CCA GAA GAT GTG; GAPDH-F, GCAAGTTCAACGGCACAG, and GAPDH-R, CCATGGTGGTGAAGACGCCA.

### Measurement of TfR1 and Fn levels in the primary sensory cortex, thalamus, and hippocampus by enzyme-linked immunosorbent assay (ELISA)

Hind limb sensory cortex, hippocampus, and thalamus tissues were collected from five rats in each group and homogenized in radioimmunoprecipitation assay buffer containing protease and phosphatase inhibitors and phenylmethylsulfonyl fluoride at 4 °C. The homogenate was centrifuged at 10,000 rpm for 30 min, and the supernatant was collected and stored at −70 °C. The protein content of each sample was determined with the bicinchoninic acid assay. TfR1 and Fn levels were determined by ELISA (abcam). Each sample was prepared in triplicate, and optical density values were calculated as mean ± standard deviation.

### Detection of protein expression levels of iron-regulatory protein 1 (IRP1), Fn, and lactoferrin (Lf) in the hind limb sensory cortex by Western blot

IRP1, Fn, Lf, and NF-κB protein levels in the hind limb sensory cortex of rats in each group were determined by western blotting. Briefly, 50 μg protein from brain tissue lysates were resolved by 10% non-denaturing sodium dodecyl sulfate-polyacrylamide gel electrophoresis and transferred to a nitrocellulose membrane, which was confirmed by Ponceau S staining. The membrane was blocked with skim milk powder at room temperature for 2 h, followed by overnight incubation at 4 °C with rat anti-human IRP1 (Santa Cruz Biotechnology, USA) (1:100), rabbit anti-human Fn (1:100, PLLABS), rabbit anti-rat NF-κB (1:400, abcam), rabbit anti-rat LF (Santa Cruz Biotechnology, USA) (1:200), and anti-β-actin (1:100) antibodies. After three washes with Tris-buffered saline containing 0.05% Tween 20, the membrane was incubated with horseradish peroxidase-conjugated secondary antibody (1:500) at room temperature. The enhanced chemiluminescence detection kit (Pierce, Rockford, IL, USA) was used to detect protein bands, which were analyzed using ImageJ software (National Institutes of Health, Bethesda, MD, USA).

### Measurement of superoxide dismutase (SOD) and malondialdehyde (MDA) in rat brain tissues

Hind limb sensory cortex, hippocampus, and thalamus tissues were collected from five rats in each group, weighed, and put into a −20 °C glass homogenizer, with the addition of 9× pre-cooled normal saline to prepare the tissue homogenate. Samples were centrifuged for 15 min at 3500 r/min at 4 °C, and the supernatants were collected. SOD was detected at 550 nm and MDA at 532 nm, according to the manufacturer’s instructions. SOD and MDA levels were calculated as follows: SOD content (U/mg prot) = (CA − TA)/CA ÷ 50% × total volume of reaction solution/sample volume ÷ TP (mg prot/ml), and MDA (U/mg prot) = (OD value of target tube − OD value of standard blank tube)/(OD value of standard tube − OD value of the standard blank tube) × concentration (10 nmol/ml) of standard ÷ TP (mg prot/mL).

### Statistical analysis

Statistical analysis was performed using SPSS 17.0. Results were presented as mean ± standard deviation (SD). Intergroup comparisons were analyzed by ANOVA. *P* < 0.05 was defined as statistically significant.

## Results

### Self-biting

Behaviors including scratching, biting, and excessive grooming below the injured segment (hind paws and tail) were observed in rats with SCI, but not in rats in the sham operation group. Within 1 month after operation, the number of rats with skin breakage below the level of injury in different groups is as follows: 8/15 in SCI group, 9/15 in l-arginine group, 6/15 in DFO group, and 0/15 in sham operation group. All damaged skin were sterilized and sutured after found.

### Determination of PWL

**Fig. 1:**
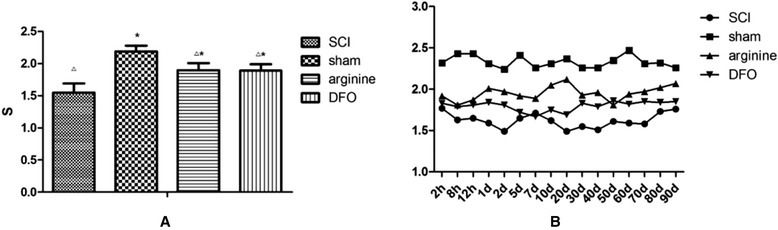
Paw-withdrawal latency (PWL) in different groups. **a** Rat PWL. *P < 0.05 compared with control group; △*P* < 0.05 compared with sham operation group. **b** Rat PWLs in different groups

Mean PWLs were 1.55 ± 0.14 s (SCI group), 2.19 ± 0.09 s (sham operation group), 1.9 ± 0.11 s (l-arginine group), and 1.89 ± 0.10s (DFO group) (Fig. 1a). The thermal pain threshold in the sham operation group was significantly higher than in the other groups (*P* < 0.05), and the threshold in the SCI group was significantly lower than in the other groups (*P* < 0.05). PWLs were higher in the sham operation group at all time points compared with the other groups, while PWLs were lowest in the SCI group (Fig. 1b).

### Brain iron levels

#### Whole-brain iron levels

**Fig. 2:**
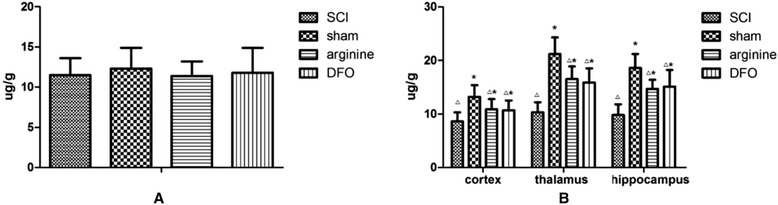
Iron levels in whole-brain and functional brain areas. **a** Whole-brain iron levels and **b** iron levels in the hind limb sensory cortex, thalamus, and hippocampus. *Compared with control group, *P* < 0.05; △compared with sham operation group, *P* < 0.05

The mean whole-brain iron levels were 11.5 ± 2.1 μg/g in the SCI group, 12.3 ± 2.6 μg/g in the sham operation group, 11.4 ± 1.8 μg/g in the arginine group, and 11.8 ± 3.1 μg/g in the DFO group (Fig. 2a). There were no significant differences in whole-brain iron levels among the groups.

#### Iron levels in functional brain areas

Iron levels in the hind limb sensory area, hippocampus, and thalamus were significantly lower in the sham operation group compared with the other groups (*P* < 0.05) and were significantly higher in the SCI group relative to the other groups (*P* < 0.05) (Fig. 2b; Table 1).

**Table 1:** Iron levels (μg/g) in the hind limb sensory cortex, thalamus, and hippocampus in different rat groups ($\overline{x}\pm \kern0.5em  s$_._)

Group	SCI	Sham	Arginine	DFO
Hind limb cortex	8.6 ± 1.7	13.2 ± 2.2	10.9 ± 1.9	10.7 ± 1.8
Thalamus	10.3 ± 1.9	21.2 ± 3.1	16.5 ± 2.4	15.9 ± 2.6
Hippocampus	9.8 ± 2.1	18.6 ± 2.6	14.7 ± 1.7	15.1 ± 3.1

### Detection of IRPs in the brain by Western blot

**Fig. 3:**
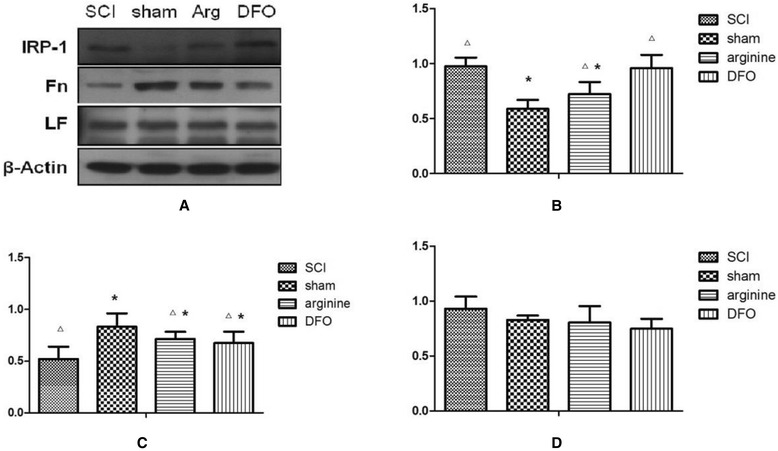
IRP1, Fn, and LF protein expression in the functional brain areas by Western blot. **a** Western blot of IRP1, Fn, and LF proteins in the hind limb sensory area of rats. **b** The gray ratio of IRP1 and the loading control β-actin were plotted. Data represent the average of three experiments. **c** Fn. **d** Lf. *Compared with SCI group, *P* < 0.05; △compared with sham operation group, *P* < 0.05

Expression levels of IRP1, Fn, and Lf proteins in the hind limb sensory area in rats in each groups are shown in Fig. 3 and Table 2. IRP1 expression was significantly lower in the sham operation group compared with the other groups (*P* < 0.05), while Fn expression was significantly higher (*P* < 0.05). In contrast, IRP1 expression levels were significantly higher in the SCI and DFO groups compared with the sham operation and arginine groups (*P* < 0.05), and Fn levels were significantly lower in the SCI group than in the other three groups (*P* < 0.05). Treatment with arginine thus reduced IRP expression and increased Fn expression in the SCI group, while DFO increased Fn expression in the SCI group but had no effect on IRP1. There were no significant differences in Lf levels among the groups.

**Table 2:** IRP1, Fn, and Lf levels in different rat groups

Group	SCI	Sham	Arginine	DFO
IRP1	0.976 ± 0.08	0.589 ± 0.08	0.723 ± 0.11	0.959 ± 0.120
Fn	0.521 ± 0.12	0.832 ± 0.13	0.715 ± 0.07	0.676 ± 0.11
LF	0.931 ± 0.11	0.828 ± 0.04	0.804 ± 0.15	0.749 ± 0.09

### TfR1 and Fn levels in the hind limb sensory area, thalamus, and hippocampus of rats by ELISA

**Fig. 4:**
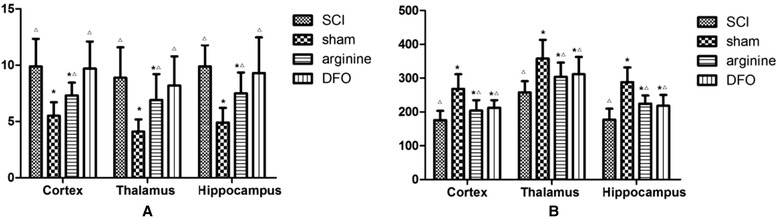
TfR1 and Fn mRNA expression in the functional brain areas by ELISA. TfR1 and Fn levels in the hind limb sensory area, thalamus, and hippocampus of rats. **a** TfR1. **b** Fn. *Compared with SCI group, *P* < 0.05; △compared with sham operation group, *P* < 0.05

TfR1 levels were significantly lower in the sham operation group compared with the other groups (*P* < 0.05), while Fn levels were significantly higher (*P* < 0.05) (Fig. 4; Tables 3 and 4). In contrast, TfR1 levels were significantly higher in the SCI group compared with the other groups (*P* < 0.05), while Fn levels were significantly lower. Arginine decreased TfR1 levels after SCI (*P* < 0.05) and increased Fn levels (*P* < 0.05), while DFO increased Fn levels (*P* < 0.05) but had no effect on TfR1 levels.

**Table 3:** TfR1 levels in different brain areas measured by ELISA

Group	SCI	Sham	Arginine	DFO
Hind limb cortex	9.9 ± 2.4	5.5 ± 1.2	7.3 ± 1.1	9.7 ± 2.4
Thalamus	8.9 ± 2.7	4.1 ± 1.1	6.9 ± 2.3	8.2 ± 2.6
Hippocampus	9.9 ± 1.9	4.9 ± 1.3	7.5 ± 1.8	9.3 ± 3.1

**Table 4:** Fn levels in different brain areas measured by ELISA

Group	SCI	Sham	Arginine	DFO
Hind limb cortex	268.2 ± 43.7	176.2 ± 27.3	204.2 ± 30.9	212.4 ± 22.7
Thalamus	358.2 ± 55.6	258.2 ± 33.0	304.2 ± 41.8	312.0 ± 51.3
Hippocampus	177.4 ± 32.3	288.6 ± 43.1	224.6 ± 24.4	218.6 ± 32.0

### TfR1 mRNA expression in the hind limb sensory area, thalamus, and hippocampus in rats by RT-PCR

**Fig. 5:**
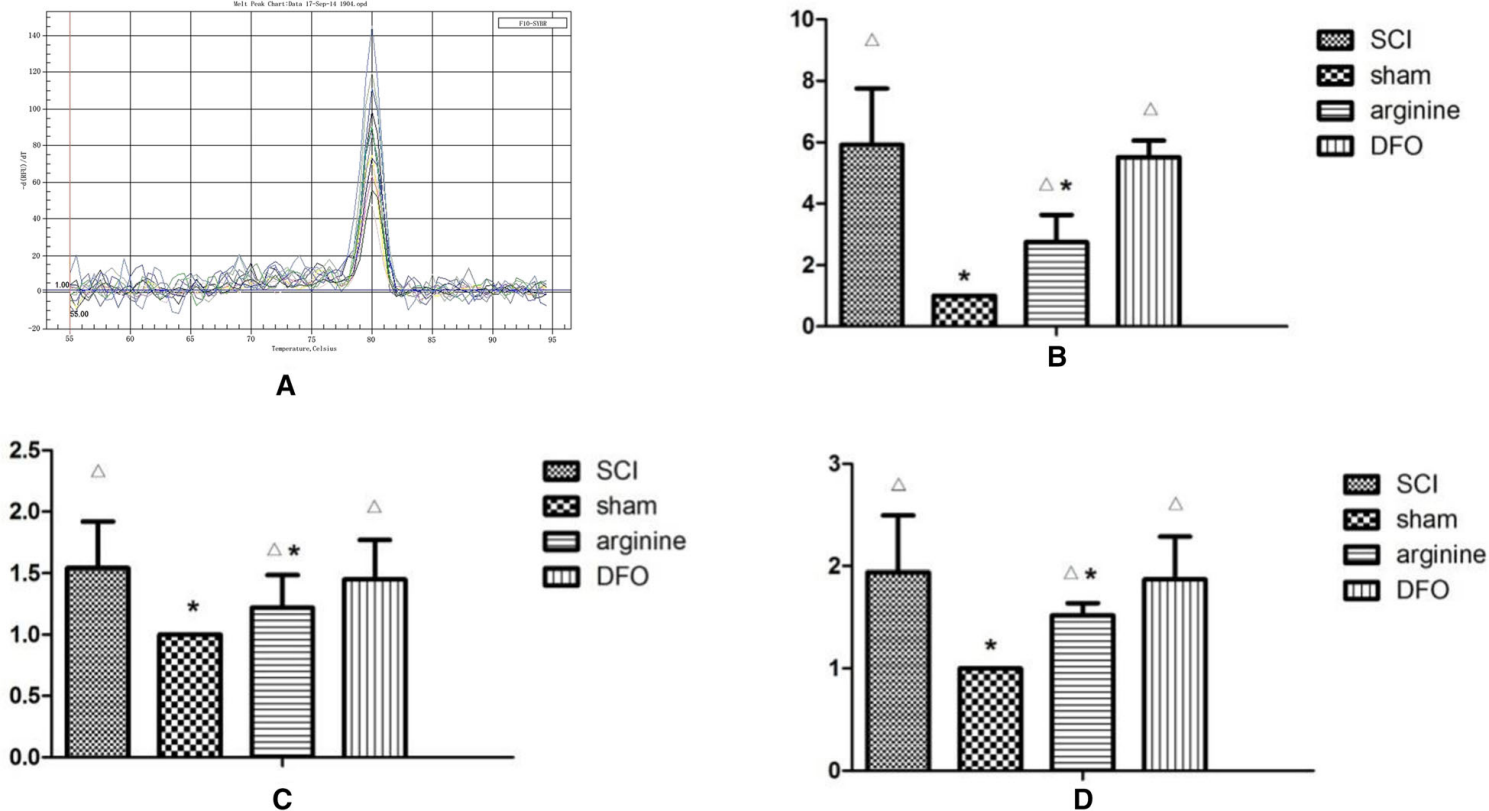
TfR1 and Fn mRNA expression in the functional brain areas by RT-PCR. **a** Melting curve of TfR1. **b** Hind limb sensory area. **c** Hippocampus. **d** Thalamus. *Compared with SCI group, *P* < 0.05; △compared with sham operation group, *P* < 0.05

TfR1 levels in the hind limb sensory area, thalamus, and hippocampus in the sham operation group were significantly lower than in the other groups (*P* < 0.05), while TfR1 levels in these areas were significantly higher in the SCI group than in the sham operation and arginine groups (*P* < 0.05) (Fig. 5; Tables 3 and 4). Arginine and DFO treatment thus reduced TfR1 mRNA levels after SCI (*P* < 0.05), with arginine having a greater effect than DFO.

### SOD and MDA levels in rat brain

**Fig. 6:**
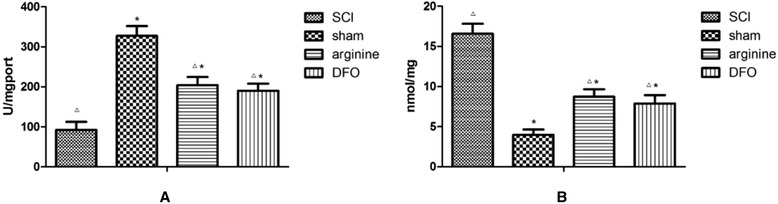
SOD and MDA levels in the functional brain areas. **a** SOD levels in rat brain. **b** MDA levels in rat brain. *Compared with SCI group, *P* < 0.05; △compared with sham operation group, *P* < 0.05

MDA levels were significantly lower in the sham operation group than in the other groups (*P* < 0.05), while SOD levels were significantly higher (*P* < 0.05) (Fig. 6; Table 5). In contrast, SOD levels were lowest and MDA levels highest in the SCI group. Arginine and DFO significantly reduced MDA and increased SOD levels (*P* < 0.05 for both).

**Table 5:** SOD and MDA levels in the hind limb sensory area in different rat groups

Group	SCI	Sham	Arginine	DFO
SOD (U/mg)	92.4 ± 20.3	327.4 ± 24.3	204.6 ± 19.8	190.3 ± 17.6
MDA (nmol/mg)	16.57 ± 1.26	3.97 ± 0.67	8.73 ± 0.94	7.88 ± 1.05

## Discussion

### Central pain

CP, also referred to as dysesthetic pain syndrome or central dysesthesia syndrome, is a stubborn and common complication of SCI, which may develop spontaneously or may be induced by skin irritation [16, 17]. The incidence of CP was reported to be 11–94% in 2 million patients with SCI, and about one third have severe CP. Patients may experience a variety of symptoms, including knife-like pain, burning pain, tingling, radiating pain, tight pain, and cold feeling, which may seriously affect their quality of life [18]. Common treatments for CP after SCI currently include physical therapy, drugs, and surgery. However, the efficacies of these treatments are unsatisfactory, and drug treatment can lead to depression, drug addiction, and other complications [19]. It is therefore essential to develop a better understanding of the pathogenesis of the disease in order to improve its treatment.

There are several hypotheses regarding the mechanisms of SCI-associated CP, including imbalance of sensory pathways [20], imbalance of inhibitory and excitatory receptors [21, 22], problems with pattern-generating mechanisms [23], and neuroimmunological mechanisms [24]; however, these hypotheses cannot fully explain the pathogenesis of CP. Recent studies have indicated that certain brain regions, such as the somatosensory center, thalamus, and limbic system, undergo remodeling after SCI to compensate for the loss of sensory function below the injured segment, and many researchers believe that this remodeling may be a key factor in SCI-associated CP [25, 26].

Patients with spinal cord transection injuries were shown to experience pain in the region distal to the injured segment, while the severity of pain was not determined by the extent of the injury [27, 28]. We therefore aimed to seek the source of pain proximal to the injured segment.

CP includes spontaneous pain caused by injury itself, with no external stimulus, as well as pain induced by external stimuli. In this study, we established a rat SCI model using the Allen impact method [29] and found that all rats, except those in the sham operation group, demonstrated excessive grooming behaviors, such as self-biting and scratching the hind limbs, which may have been caused by spontaneous pain in the hind limbs. We also showed that the thermal pain threshold in the lower extremities was significantly decreased in the SCI/CP groups, thus triggering hyperalgesia. These results indicated that both spontaneous and induced pain were induced in this rat model, in line with previous studies [30–32]. Treatment with DFO or arginine significantly increased the thermal pain threshold and reduced excessive grooming behavior, suggesting that these drugs relieved the induced and spontaneous SCI-associated CP.

### Iron overload in the brain

Iron is an important trace element in the human body and is widely distributed in the brain, where it participates in many important physiological and biochemical processes, including DNA, RNA, and protein synthesis, myelin synthesis, myelinogenesis, and development, as well as the synthesis of some neurotransmitters such as dopamine. However, excess iron is toxic to the human body, and abnormal accumulation of iron in various brain areas has been found in many neurodegenerative disorders [33, 34]. Iron is known to induce the production of hydroxyl radicals through the Fenton reaction [35] (Fe2++ H2O2 → Fe3 + ·OH + OH), thus exacerbating oxidative stress and leading to tissue and cell damage.

Iron is redistributed and deposited in certain brain areas in some neurodegenerative diseases, such as Alzheimer’s disease, Parkinson’s disease, Huntington’s disease, and Hallervorde–Spatz syndrome [36–38], and iron levels have also been shown to be increased in brain areas in animals under stress conditions, such as heat stress, exercise stress, and seasickness [39, 40]. Although increased iron levels have not been conclusively identified as either the cause or consequence of these diseases, recent studies of genes associated with iron metabolism in the brain suggest that increased iron is an initiating factor of neuronal death in some neurodegenerative diseases [41].

In the present study, we measured iron levels in whole brain and specific brain areas of rats by atomic absorption spectrophotometry and found no significant differences in whole-brain iron levels among the control, SCI, and SCI + l-arginine- or SCI + DFO-treated rats. However, iron levels in the thalamus, hippocampus, and hind limb sensory area were significantly increased in all SCI/CP groups, with an iron-deposition distribution similar to that in some neurodegenerative diseases. The hind limb sensory area is the projection area of the hind limb in the cortex, while the thalamus and hippocampus are transit areas of sensory transduction. Lesions in these areas can cause CP [42, 43]. In the current study, iron levels in certain brain areas remained elevated 12 weeks after surgery to induce SCI, supporting long-term deposition of iron in the brain consistent with the long duration of CP.

The fact that iron levels increased in certain brain areas but not in the brain as a whole may be attributable to the function of the blood–brain barrier, which isolates the brain from the external environment. Although iron levels increased in some areas, they remained stable overall, consistent with the results of previous studies [44].

### Mechanisms of iron overload in the brain

Maintenance of iron homeostasis in the brain depends on the normal expression and coordination of a variety of brain iron-metabolism proteins. Penetration of iron through the blood–brain barrier and the uptake of iron by neurons are mainly mediated by the classic Tf/TfR pathway [45–48].We found that TfR1 mRNA and protein levels were increased in the thalamus, hippocampus, and hind limb sensory areas of SCI/CP rats compared with the sham operation group, suggesting that iron overload in these brain areas may be caused by increased iron levels mediated by the Tf/TfR pathway.

Fn is a natural iron chelator that is widely expressed in neurons and glial cells in human and rodent animal brains. Iron stored by Fn accounts for 75% of total brain iron [49]. The structure of Fn includes H and L subunits, of which the L subunit is associated with long-term iron storage [50, 51]. Ferrous iron taken up by tissue cells is oxidized to ferric iron and sequestered by Fn and stored in a non-toxic form. [52] Previous studies have shown that upregulation of Fn may limit iron-induced brain injury [53]. Furthermore, Fn was found not to increase in line with increasing iron levels in Parkinson’s disease and Alzheimer’s disease [54], suggesting that free iron may not be captured and stored by Fn, and peroxidation caused by free-iron toxicity may not be prevented.

In this study, levels of the Fn in the thalamus, hippocampus, and hind limb sensory area were significantly decreased in SCI/CP rats compared with the sham operation group, suggesting that the iron-storage capacity was reduced in these areas, potentially leading to increased free iron.

Tfr and Fn play a crucial role in maintaining iron homeostasis in the brain. Expression levels of these two proteins are mainly regulated by the iron-responsive element (IRE)/IRPs. In the absence of iron, IRPs bind to IREs, which upregulate TfR1 synthesis and downregulate Fn synthesis. Iron deficiency thus leads to increased iron uptake and reduced iron storage. In contrast, when iron is in excess, IRPs do not bind to IREs, TfR1 synthesis is downregulated, and Fn synthesis is upregulated, leading to decreased iron intake and increased iron storage. However, IRP activity is not only regulated by intracellular iron content. Previous studies found that the IRE/IRP complex was more stable in the brain of patients with Alzheimer’s disease. Increased stability of this complex can stabilize Tfr mRNA levels and decrease Fn synthesis, thereby increasing iron uptake and decreasing iron storage in the brain in these patients, resulting in brain iron overload and the induction of oxidative stress and neuronal apoptosis.

The results of the current study showed that IRP1 protein levels were significantly upregulated in the hind limb sensory area, thalamus, and hippocampus of SCI/CP rats, compared with the sham operation group, while TfR was increased and Fn was decreased in these areas. This suggests that iron regulation was disrupted in these areas after SCI, and that IRP1 may be the initiating factor for this process. In addition to the Tf/TfR pathway, human Lf/Lf receptor may also play a role in iron transport through the blood–brain barrier. However, we found no differences in Lf expression in the thalamus, hippocampus, and hind limb sensory area among the different rat groups, suggesting that Lf-mediated iron uptake may not be involved in iron overload after SCI.

DFO is a potent iron ion chelating agent, which has been shown to penetrate the blood–brain barrier rapidly and accumulate in the brain parenchyma. It can chelate free iron ions to form a relatively stable compound and effectively prevent the release of iron ions from Fn, thus significantly reducing peroxidation damage caused by iron overload [23]. In our study, DFO significantly reduced iron levels in the hippocampus, hind limb sensory cortex, and thalamus, and increased Fn levels in these areas, suggesting that DFO may chelate the iron in these areas and inhibit the degradation of Fn. However, whole-brain iron levels were not reduced, suggesting that DFO decreased iron levels by binding iron ions and inhibiting the formation of free iron, rather than by promoting the excretion of iron outside the brain.

### Iron overload and oxidative stress

There are two types of free radical scavengers in the human body, antioxidases and antioxidants, which provide electrons to reduce and block the formation of oxygen free radicals and thus prevent cell damage. Free radicals can induce scavenger enzymes to maintain a dynamic balance [55]. The central nervous system is rich in lipids, and free radicals can cause lipid peroxidation and induce pathological changes in cell morphology and function. Arachidonic acid substances generated by lipid peroxidation, including prostaglandins D2, E2, and I2 and leukotrienes, can act on the nerve and glial cells to injure cell membranes and cause cell dysfunction [56].

SOD and MDA reflect the degree of lipid peroxidation. MDA is a metabolite of the peroxidation reaction of membrane unsaturated fatty acids induced by oxygen free radicals, and an indicator of the degree of cell damage [57]. SOD is a natural antioxidase capable of cleaning oxygen radicals, thereby blocking the lipid peroxidation chain reaction, and providing an indicator of the body’s ability to scavenge oxygen free radicals. Previous studies found that iron overload increased serum and organ levels of MDA and reduced SOD [58, 59]. Given that increased brain iron levels and accompanying oxidative stress have been identified in many neurodegenerative diseases, some researchers have suggested that abnormal increases in brain iron may lead to the generation of large numbers of free radicals and further induce cell death, which may be one reason for the observed neuronal death in neurodegenerative diseases. The addition of ferrous sulfate in the diet has been shown to increase iron ion levels and oxygen free radicals in rat brains, and to cause neuronal injury, and even death [12]. Following cerebral hemorrhage and cerebral ischemia reperfusion, excessive iron ions can catalyze lipid peroxidation to produce oxygen free radicals, which attack cell proteins and nucleic acids leading to peroxidation injury, which represents an important mechanism of brain injury secondary to cerebral hemorrhage [60–64]. The potential causative role of oxidative stress caused by iron overload in the brain in neurodegenerative diseases is supported by the abnormal accumulation of iron in certain brain areas in patients with Parkinson’s disease and Alzheimer’s disease [65].

In the present study, SOD activity was significantly decreased and MDA content was significantly increased in brain tissues of rats with CP. This suggests that large numbers of oxygen free radicals were produced in the brain, and that decreased SOD activity caused lipid peroxidation injury of biomembranes, which increased the content of MDA. Intervention with DFO decreased iron levels and restored SOD activity, associated with decreased MDA content.

## Conclusions

The results of this study suggest that SCI may trigger glucocorticoids to activate nitric oxide synthase and further activate IRP/IRE, leading to intracranial iron overload. Iron overload may in turn cause neuronal injury via the oxidative stress signaling pathway, resulting in neuronal damage or loss, and eventually leading to central pain. Treatment with iron chelators or nitric oxide synthase inhibitors may effectively alleviate central pain after SCI.

## Abbreviations

CP: Central pain
SCI: Spinal cord injury
DFX: Deferoxamine
NOS: Nitric oxide synthase
TfR1: Transferrin receptor 1
Fn: Ferritin
Lf: Lactoferrin
IRP1: Iron-regulatory protein1
SOD: Superoxide dismutase
MDA: Malondialdehyde
IRE: Iron-responsive element
